# Non-syndromic Cleft Palate: An Overview on Human Genetic and Environmental Risk Factors

**DOI:** 10.3389/fcell.2020.592271

**Published:** 2020-10-20

**Authors:** Marcella Martinelli, Annalisa Palmieri, Francesco Carinci, Luca Scapoli

**Affiliations:** ^1^Department of Experimental, Diagnostic and Specialty Medicine, Alma Mater Studiorum – University of Bologna, Bologna, Italy; ^2^Department of Morphology, Surgery and Experimental Medicine, University of Ferrara, Ferrara, Italy

**Keywords:** non-syndromic cleft palate, NSCPO, risk factors, etiology, *GRHL3*, *FOXE1*, *PAX7*

## Abstract

The epithelial and mesenchymal cells involved in early embryonic facial development are guided by complex regulatory mechanisms. Any factor perturbing the growth, approach and fusion of the frontonasal and maxillary processes could result in orofacial clefts that represent the most common craniofacial malformations in humans. The rarest and, probably for this reason, the least studied form of cleft involves only the secondary palate, which is posterior to the incisive foramen. The etiology of cleft palate only is multifactorial and involves both genetic and environmental risk factors. The intention of this review is to give the reader an overview of the efforts made by researchers to shed light on the underlying causes of this birth defect. Most of the scientific papers suggesting potential environmental and genetic causes of non-syndromic cleft palate are summarized in this review, including genome-wide association and gene–environment interaction studies.

## Introduction

Orofacial clefts are the most common orofacial malformations in humans and include cleft lip (CL), cleft lip with or without cleft palate (CL/P), and cleft palate only (CPO). CPO (MIM 119540) is a birth defect that occurs when only the secondary palate is involved and can affect the hard palate and/or the soft palate, sometimes limited to one cleft uvula. It represents one third of all oral clefts and affects about 1 to 25 per 10.000 newborns worldwide (Mossey et al., 2009). The incidence of CPO is highly influenced by ethnicity and race, with the highest rates observed in non-Hispanic Whites and the lowest in Africans (Mossey and Modell, 2012). Besides, females are more prone to the defect than males (1:1.075) (Mossey and Catilla, 2003).

Cleft palate only is a multifactorial disorder influenced by both genetic and environmental factors that act during palatogenesis (Meng et al., 2009; Dixon et al., 2011). Moreover, the local changes in growth factors, extracellular matrix (ECM), and cell adhesion molecules may also play a part in CPO onset. A variety of signaling pathways are involved in palate development and several mutations on developmental genes that have been found to contribute to CPO will be discussed.

Furthermore, the environmental contribution to CPO by tobacco, alcohol and multivitamin supplementation has been highlighted by epidemiological studies hereinafter detailed.

### Classification

**FIGURE 1:**
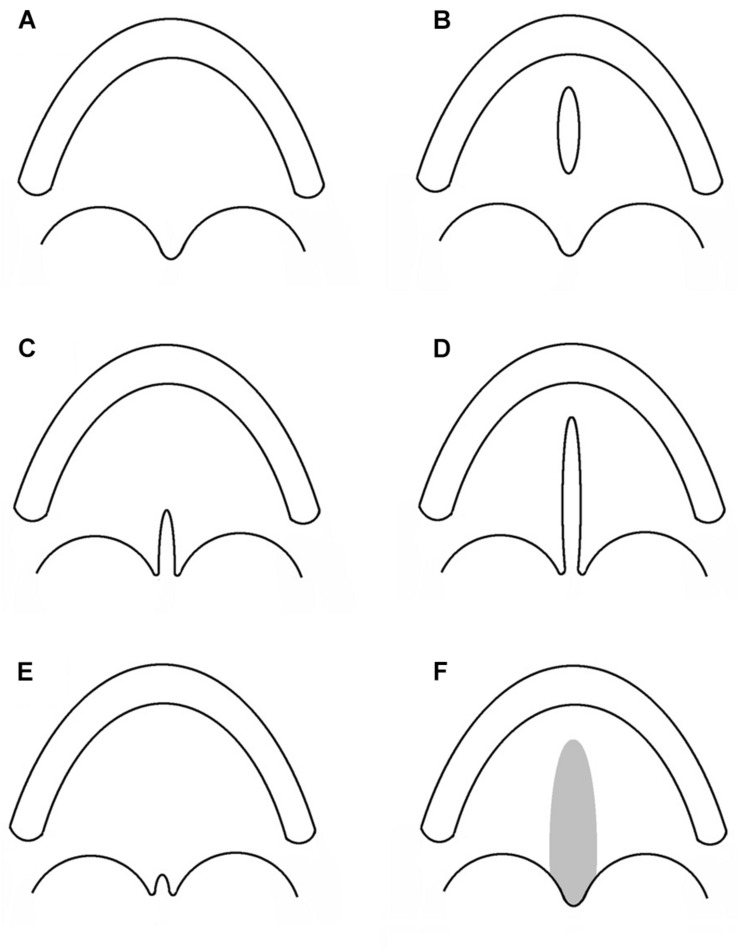
Subtypes of cleft palate only. Each sketch illustrates the alveolar process and the palatal rim. **(A)** Normal palate. **(B)** Cleft of the hard palate. **(C)** Cleft of the soft palate. **(D)** Cleft of the hard and soft palate. **(E)** Cleft uvula. **(F)** Submucous cleft palate.

Cleft lip with or without cleft palate and CPO are considered as different congenital malformations, having distinct embryologic origins and recurrence risks. Hence, CPO refers to any cleft of the palate which is posterior to the palatine foramen, and which does not involve the alveolar process or lip. A number of different classifications have been proposed since Veau’s version in 1931 (Veau, 1931; Danescu et al., 2015). In Figure 1, a collection of different types of CPO, including the recently introduced submucous microform (Danescu et al., 2015).

Another kind of classification divides CPO into two major groups: (i) isolated CPO or non-syndromic CPO (NSCPO), when clefting is an isolated defect unassociated with any other additional anomalies, representing 54.8% of the total CPO cases; (ii) syndromic CPO, when clefting is associated with other anomalies in a recognizable (27.2%), or unrecognizable syndrome (18%) (Calzolari et al., 2004).

Although the presence of an evident pattern of anomalies makes the diagnosis easier, some features may not be fully expressed or could represent subclinical phenotypes more difficult to diagnose. Most frequently, the defects associated with CPO affect the heart (31.1%), the encephalon (hydrocephaly 11.2%), the urinary tract (9.7%), or digits (polydactyly 9.2%) (Mossey and Catilla, 2003). The number of syndromes involving cleft palate, among orofacial clefts, is subject to progressive implementation but already in 1978 it was quoted as 77 (Cohen, 1978).

The most common syndromes in which cleft palate deformity represents one of the features are: Apert Syndrome, Crouzon Syndrome, DiGeorge Syndrome, Loeys-Dietz Syndrome, Treacher Collins Syndrome, X-linked cleft palate Syndrome, Ankyloglossia, Bamforth-Lazarus Syndrome^1^. An exhaustive overview of the syndromes that count cleft palate among their features, as well as the responsible genes, can be found in a paper by Burg et al. (2016).

### Palatogenesis

The human palate separates the nasal and oral cavities and consists of a bony *hard palate* (the anterior two thirds) and a fibromuscular *soft palate* (the posterior one third). The incisive foramen divides the *hard palate* into primary and secondary portions. The secondary portion separates the nasal passage from the pharynx and is marked by a median palatine raphe. The *soft palate*, or velum, is a mobile muscular fold forming a curtain posterior to the hard palate, ending with a conical mass called the *uvula*. The soft palate separates the nasopharynx from the oropharynx.

Palatal development takes place between the fifth and twelfth embryonic weeks, with the most critical period occurring during the sixth to ninth weeks (Merritt, 2005), when the lateral palatine processes fuse, after the fusion of the secondary palate to the primary palate with a junction marked by the incisive foramen. The palate body is composed by mesenchymal cells, mainly derived from migrating neural crest cells, layered by epithelial cells that surround the mesenchyme (Ito et al., 2003). The correct growth, migration, transition, differentiation, and apoptosis of these cells form the basis of a regular development of the palate. Palatal shelves, initially grown in a vertical position, have to rotate from the sides to the top of the tongue in order to acquire a horizontal position. Then, shelves approach and fuse with each other in an antero-posterior direction in the transient midline epithelial seam (Ferguson, 1988; Meng et al., 2009).

Conceivably, there are several molecular, mechanical and morphological steps, including the key step of epithelial to mesenchymal transition, involved in the orchestra of palate development which could go wrong (Diewert and Lozanoff, 2002). Clefting of the secondary palate may then arise in any one of these critical steps, and may be caused by failure in growth, elevation, adhesion or fusion of the palatal shelves.

### Epidemiology

Cleft palate affects newborns with a world prevalence of about 3.1/10,000, which significantly varies depending on the geographical location. According to the Global Burden of Disease (GBD) study, the highest rates of CPO are found in the Oceanic continent, and the lowest in Sub-Saharan Africa, with overall values ranging from 0.4 to 11.3 every 10,000 births (Mossey and Modell, 2012). Nevertheless, there are very few epidemiological studies about OFC in African populations and estimated values of CPO incidence may not be accurate. Moreover, even inside the same continent, CPO prevalence values vary among different geographical areas, as in Europe. In fact, according to the EUROCAT registries Finland and Malta show the highest rates while Portugal and Romania the lowest, with more than three-times difference in rates (EUROCAT).

Besides geographical differences, there is also a disparity in the CPO incidence between genders. In fact, while for isolated CL/P cases the sex ratio was estimated at 1.81 (95% CI, 1.75–1.86), isolated CPO counts more females than males, with a sex ratio of 0.93 (95% CI, 0.89–0.96) in the world (Mossey and Catilla, 2003) and reported as 0.78 in Europe (Calzolari et al., 2004). This discrepancy is probably due to the delay of 1 week in the fusion of the palatal shelves in female embryos compared to males (Burdi and Faist, 1967). Possibly, in this period of time the morphogenesis could be more influenced by teratogens, thus justifying the higher CPO rate in girls.

## Etiology

### Environmental Factors

The complex etiology of NSCPO can be explained, beside genetic factors, by the intervention of unmodifiable (e.g., race/ethnicity, sex, family history of clefts) and modifiable factors, acting in the period from 1 month before through 2 months after conception. Fundamentally, maternal factors such as health/disease status, lifestyle, medication, exposure to environmental teratogens can affect/influence the intrauterine environment, more heavily during embryo development, and have been the topic of a number of studies looking at their association with NSCPO risk.

Surprisingly, it is the older age of the father that has been reported to increase the risk of NSCPO in the offspring, rather than that of the mother (Savitz et al., 1991; Bille et al., 2005) as confirmed by a meta-analysis (Herkrath et al., 2012).

Among the maternal behavioral factors, alcohol consumption during the first trimester of pregnancy has been suggested to have a correlation with oral cleft onset (Romitti et al., 2007; DeRoo et al., 2008). However, there are few evidences supporting a role in the CP etiology (DeRoo et al., 2008; Sabbagh et al., 2015). A recent meta-analysis (Yin et al., 2019) concluded that there is no evidence of a correlation between moderate periconceptional alcohol consumption and the risk of both non-syndromic CL/P and CP in infants. However, authors suggest that the potential risk of binge-drinking cannot be excluded, because of the heterogeneity of the threshold level for alcohol consumption in the different investigations and of the limited sample sizes of heavy drinker mother cohorts.

Also maternal tobacco smoking has been deeply investigated as an NSCPO risk factor. The studies were recently considered in two meta-analyses, which concluded by supporting a role for both maternal tobacco smoking (Little et al., 2004) and passive smoking (Sabbagh et al., 2015) in NSCPO etiology. Nevertheless, it should be taken into account that the results of meta-analyses could be biased because the sample size, ethnicity, and consumption levels vary widely from one study to another. Moreover, the alcohol and tobacco teratogenic dose-response effect is still a matter of debate.

A recent study examined the effect of maternal exposure to water disinfection by-products (DBP), a heterogeneous group of compounds that can originate from a combination of chemical disinfectants (e.g., chlorine, chloramine) and organic matter present in water (Kaufman et al., 2018). Studying the association between DBP and the risk of craniofacial malformation, the authors obtained evidence that some DBPs (specifically: DBP9, HAA5, trichloroacetic acid, and dichloroacetic acid) can increase the risk of NSCPO. Similarly, a previous study suggested an increased risk of NSCPO as a result of maternal exposure to total trihalomethanes, but at lower ranges than those of the above-mentioned work (Hwang et al., 2008). Moreover, nitrate intake from drinking water was seen to increase the risk of NSCPO in a large cohort study (OR, 1.9; 95% CI, 1.17–3.09) (Brender et al., 2013).

Since the nutritional status of the embryo is fully dependent on maternal food intake and metabolism, unbalanced maternal nutrition during the first trimester of pregnancy can lead to birth defects. Several essential micronutrients, substances that cannot be synthesized by our body in sufficient amount, are needed for health maintenance, pregnancy progression and normal embryonic development. In case of maternal nutrition deficiencies, supplementation during pregnancy is strongly advised. It is commonly accepted that folic acid-fortified multivitamin supplementation before conception and continuing through the first trimester reduces the overall occurrence of several congenital anomalies. However, only few systematic reviews or meta-analyses regarding orofacial clefts have been conducted and findings on cleft palate are inconclusive. A systematic review analyzed data from five clinical trials in order to evaluate if supplementation with folic acid, alone or in combination with vitamins and minerals, can prevent the occurrence of neural tube defects (NTD) and other birth defects. No evidence of any preventive or negative effects on cleft palate by periconceptional oral folate supplementation was detected (RR, 0.73; 95% CI, 0.05–10.89) (De-Regil et al., 2015). A reduced risk of NSCPO resulted in a meta-analysis of case–control studies for mothers who took multivitamin supplementation starting from before pregnancy (OR, 0.76; 95% CI, 0.62–0.93) (Ingrid Goh et al., 2006). An observational study reported that after food grain fortification with folic acid, the prevalence of NSCPO in United States had a significant 12% reduction (Canfield et al., 2005). On the other hand, Sutton et al. (2011) measured levels of red cell folate (RCF), vitamin B12 and homocysteine (tHcy) in the blood of women who were pregnant with a malformed baby at a time when multivitamin supplementation or food fortification was still rare. They found that the level of B12, which plays a critical role in folate metabolism, was surprisingly higher in mothers expecting a baby with cleft palate than in mothers of unmalformed offspring, with no differences in levels of RCF and tHcy (Sutton et al., 2011).

Retinoic acid (RA), a derivate of Vitamin A, is an important regulator of processes that occur during embryogenesis, such as proliferation, differentiation, and apoptosis (Finnell et al., 2004). Only few epidemiological studies on human maternal intake of Vitamin A are available (Ackermans et al., 2011). Nevertheless, an excess of Vitamin A is considered teratogenic, causing congenital malformations, including cleft palate. On the other hand, Johansen et al. (2008) evidenced a substantial protective association between high (1.91–9.64 mg) maternal intake of Vitamin A and risk of NSCPO (adjusted OR [aOR], 0.47; 95% CI, 0.24–0.94), indicating that a deficit of Vitamin A is also to be considered a risk factor for NSCPO. The need for maternal zinc supplementation during pregnancy is still an unresolved issue with conflicting results from observational data. Higher maternal plasma zinc concentration was seen to be protective against NSCPO in a sample study of Filipino women (Tamura et al., 2005), whereas the same authors did not confirm the association in a sample study from Utah (Munger et al., 2009), probably attributable to the fact that zinc status in US mothers is not compromised to a certain severity as commonly seen in the Philippines.

Medicament intake during the periconceptional/first trimester period has long been generally ascertained to be correlated with an increased risk of adverse maternal outcomes. On the spectrum of congenital anomalies considered, cleft palate showed a significant increase in odd ratio with several treatments, including the use of inhaled β2-agonists as a medical treatment for asthma (OR, 1.63; 95% CI, 1.05–2.52) (Garne et al., 2015); use of valproic acid (OR, 5.8; 95% CI, 3.3–9.5) and carbamazepine (OR, 2.4; 95% CI, 1.1–4.5) as antiepileptic drugs (in the United States) (Gilboa et al., 2011); use of aspirin as analgesic (OR, 1.7; 95% CI, 1.0–2.9) (Hernandez et al., 2012); use of corticosteroids (OR, 5.3; 95% CI, 1.1–26.5) (Carmichael and Shaw, 1999); use of ondansetron to treat hyperemesis gravidarum (RR, 1.29; 95% CI, 1.00–1.65) (Huybrechts et al., 2018); and the use of nitrosatable amides as anti-infectives (aOR, 1.27, 95% CI, 1.00–1.62) (Brender et al., 2012).

Maternal sickness could influence the pregnancy outcome, as hypothesized for influenza, common cold and cystitis by Metneki et al. (2005). These authors highlighted the teratogenic effect of hyperthermia, which was confirmed the following year by Acs et al. (2006), who reported that the fever associated with acute respiratory infections seemed to increase the risk for posterior cleft palate in their Hungarian sample study. In 2020, in a Caucasian population-based case–control study with 751 NSCPO cases, again by Acs et al. (2020), analyzed the role of maternal diseases in the increased risk for developing NSCPO. Specifically, the authors evidenced significantly altered odd ratios for CP when mothers where affected by acute inflammatory diseases such as: influenza (OR, 1.8; 95% CI, 1.3–2.5), acute upper respiratory infections (OR, 2.5; 95% CI, 1.9–3.1), acute lower respiratory infections (OR, 2.4; 95% CI, 1.4–4.2), urogenital infections (OR, 2.0; 95% CI, 1.4–2.8), and unspecified high temperature (OR, 8.1; 95% CI, 2.9–22.6). Besides, they found a strong association with herpes simplex infection (OR, 14.8; CI, 5.7–38.5). The association between chronic maternal diseases and an increased risk for NSCPO in the offspring was observed for: Graves’ disease (OR, 4.3; 95% CI, 1.7–10.6), epilepsy (OR, 4.6; 95% CI, 2.4–8.8), migraine (OR, 2.8; 95% CI, 1.2–6.8), essential hypertension (OR, 1.7; 95% CI, 1.2–2.4) and neuro-musculoskeletal pain syndromes. However, these findings have to be considered along with the teratogenic effect of medicament assumption to alleviate the symptoms of such conditions (Acs et al., 2020). According to Acs et al. (2020) the significantly higher risk observed for anemic mothers to have an NSCPO child (OR, 1.84; 95% CI, 1.25–2.71) could be a consequence of embryonic hypoxia during the critical morphogenetic period.

A meta-analysis carried out to assess the association between maternal obesity and congenital anomalies, evidenced a barely but significantly increased risk of cleft palate (Stothard et al., 2009), a datum that was later confirmed by Block et al. (2013).

Gestational diabetes mellitus has been associated with an increased risk of cleft palate in the offspring (OR, 1.54; 95% CI, 1.01–2.37) of women with a pre-pregnancy BMI ≥ 25 kg/m^2^, but the authors stress the need to consider the possibility of undiagnosed type 2 diabetes mellitus and the subsequent hyperglycemia as a real cause of the embryo malformation (Correa et al., 2008). With regard to pregestational diabetes, a very large population-based birth defect case–control study, carried out this year in the United States, evidenced an association with cleft palate alone (OR, 4.3; 95% CI, 2.9–6.5), while a smaller increased risk was observed in association with gestational diabetes (OR, 1.4; 95% CI, 1.1–1.8) (Tinker et al., 2020).

### Genetic Factors

Increased recurrence risk in relatives indicates a high level of heritability for NSCPO (Grosen et al., 2010). Hence, the evidence of a genetic component in the etiology of this birth defect and the challenge taken up by researchers to obtain a better understanding of the NSCPO molecular bases.

During secondary palate formation, the palatal shelves grow, approach and fuse. The cells need to activate a series of biological mechanisms, including cell migration, epithelial–mesenchymal transition, and apoptosis, necessary to remove epithelial cells from the palatal epithelia medial edge, leading to the continuity of the mesenchyme and to palatal formation. For this reason, it has been hypothesized that many genes that code for proteins involved in the formation of cytoskeleton filaments, for cell adhesion molecules, or for ECM components, may contribute, if altered, to the clefted palate phenotype (Gibbins et al., 1999; Tudela et al., 2002). Here, we browse through the association studies on genes involved in mechanisms crucial for palatal development (Table 1).

**TABLE 1:** Published association studies between gene polymorphisms and NSCPO.

**Gene^#^**	**Locus**	**SNP information**	**Country**	**Sample size**	***P*-value**^†^	**OR (95% CI)**	**References**
*CDH1*	16q22.1	rs16260	China	26 cases – 107 controls	0.004	6.90 (1.47–32.40)^‡^	Song and Zhang (2011)
		rs11642413	Iran	31 cases – 100 controls	0.019	3.70 (1.26–10.87)	Rafighdoost et al. (2013)
		rs16260		31 cases – 100 controls	NS		
		rs9929218	Latvia	10 cases – 190 controls	NS		Krasone et al. (2014)
		rs16260; rs11642413	Africa	163 cases – 1078 controls	NS		Gowans et al. (2016)
		rs1801552	China	115 cases – 271 controls	0.036	0.62 (0.40–0.97)	Song et al. (2017)
		rs16260; rs9929218			NS		
*COL2A1*	12q13.11	rs1793949	Baltic regions	104 cases – 606 controls	7.26 × 10^–4^	1.659 (1.235–2.229)	Nikopensius et al. (2010)
		rs6823			0.0058	1.517 (1.270–2.041)	
		rs12228854			0.0067	1.663 (1.148–2.409)	
		rs12368284			0.0093	0.661 (0.483–0.904)	
		rs10875713			0.0197	1.596 (1.074–2.370)	
		rs11168359			0.0203	0.544 (0.323–0.916)	
		rs1793949	Brazil	107 triads	NS		Machado et al. (2016)
*CRISPLD2*	16q24.1	rs12051468; rs8061351; rs721005; rs1546124; rs16974880; rs4783099	Ireland	293 cases – 902 controls	NS		Carter et al. (2010)
		rs1546124	China	118 cases – 463 controls	5.4 × 10^–4^	2.93 (1.69–5.07)^‡^	Shen et al. (2011)
		rs4783099			1.1 × 10^–3^	0.48 (0.30–0.77)	
		rs16974880			NS		
		rs4783099	Africa	163 cases – 1078 controls	0.02	0.74	Gowans et al. (2016)
		rs1546124			NS		
		rs4783099	Brazil	236 cases – 693 controls	0.01	1.31 (1.05–1.62)	Messetti et al. (2017)
		rs1546124; rs8061351; rs2326398			NS		
*FOXE1*	9q22.33	rs1867278	Denmark, Norway, United States, Philippines	524 triads	4.1 × 10^–4^		Moreno et al. (2009)
		Other 14 SNPs genotyped			NS		
		rs111846096	Thailand	77 cases – 90 controls	NS		Srichomthong et al. (2013)
		rs4460498	Germany, The Netherlands, Maya	165 cases – 1500 controls	0.017	0.81 (0.56–1.17)	Ludwig et al. (2014)
			Europe	156 triads	0.043	0.913 (0.51–1.65)	Ludwig et al. (2014)
		rs4460498; rs3758249	China	51 triads	NS		Liu et al. (2015)
		rs894673; rs3758249	Africa	163 cases – 1078 controls	NS		Gowans et al. (2016)
		rs6586	California (Hispanic)	66 cases – 476 controls		0.34 (0.13–0.90)^‡^	Lammer et al. (2016)
		rs4618817				0.34 (0.15–0.80)	
		other 11 SNPs genotyped			NS		
*GRHL3*	1p36.11	rs2486668; rs545809	China	297 cases – 377 controls	NS		He and Bian (2016)
		rs41268753	Norway, Denmark, United States	246 cases – 1685 controls	2.81 × 10^–4^	2.16 (1.43–3.27)	Leslie et al. (2016)
		rs113965554			6.82 × 10^–4^	1.97 (1.33–2.91)	
		rs41268753	Europe, Yemen	288 cases – 725 controls	2.63 × 10^–5^	2.46 (1.62–3.74)	Mangold et al. (2016)
			Germany	116 cases^§^ – 267 controls	0.94		Mangold et al. (2016)
*IRF6*	1q32.2	rs4844880; rs669694; rs2235371; rs2235375; rs2013162; rs126280	Norway	117 triads	NS		Jugessur et al. (2008)
		rs2235371	China	25 cases – 96 controls		0.25 (0.061–0.57)	Tang et al. (2009)
		rs2235371; rs2013162; rs7552506; rs2235377	Ireland	293 cases – 902 controls	NS		Carter et al. (2010)
		rs17389541	Baltic regions	104 cases – 606 controls	5.45 × 10^–4^	1.726 (1.263–2.358)	Nikopensius et al. (2010)
		rs9430018			0.0454	1.351 (1.006–1.814)	
		rs4844880; rs2235371; rs2013162; rs861019; rs2073487; rs658860	Brazil	53 cases – 285 controls	NS		Letra et al. (2012)
		rs34743335; rs642961	Africa	163 cases – 1078 controls	NS		Gowans et al. (2016)
		rs642961; rs77542756; rs2235371	Thailand	83 triads	NS		Wu-Chou et al. (2019)
		rs2235371	Brazil	38 cases – 182 controls	0.004	3.01 (0.97–8.97)	Bezerra et al. (2019)
		rs642961; rs2236907; rs861019; rs1044516			NS		
*JAG2*	14q32.33	rs11624283	Estonia	53 cases – 205 controls	0.0016		Jagomagi et al. (2010)
		rs10134946	Baltic regions	104 cases – 606 controls	0.0318	1.384 (1.028–1.864)	Nikopensius et al. (2010)
		rs1057744	Brazil	81 cases – 413 controls	NS		Paranaiba et al. (2013)
*MSX1*	4p16.2	rs1106514	Estonia	53 cases – 205 controls	0.0037		Jagomagi et al. (2010)
			Baltic regions	104 cases – 606 controls	0.0095	1.482 (1.100–1.998)	Nikopensius et al. (2010)
		rs3821949; rs12532; rs104893854 – P147Q	China	42 triads	NS		Huang et al. (2011)
		rs62636562	Brazil	81 cases – 413 controls	NS		Paranaiba et al. (2013)
		rs12532	Iran	31 cases – 100 controls	0.008	10.83 (2.38–49.38)^‡^	Rafighdoost et al. (2013)
		rs3775261			NS		
		rs12532; rs3821949	China	56 cases – 605 controls	NS		Ma et al. (2014)
		rs2073242	Brazil	75 cases – 823 controls	NS		Kuchler et al. (2014)
		rs1106514	Brazil	107 triads	NS		Machado et al. (2016)
		rs115200552	Africa	163 cases – 1078 controls	0.01	1.81	Gowans et al. (2016)
		rs12532			NS		
		pCA	Europe	180 triads	NS		Mossey et al. (2017)
*PAX7*	1p36.13	rs742071	Europe	266 triads	NS		Bohmer et al. (2013)
		rs4920520; rs766325	Iowa, Asia	94 triads	NS		Butali et al. (2013)
		rs742071	China	56 cases – 605 controls	NS		Pan et al. (2013)
		rs766325; rs742071	Africa	163 cases – 1078 controls	NS		Gowans et al. (2016)
		rs742071	China	144 triads	0.025	3 (1.09–8.25)	Duan et al. (2017)
		rs4920522; rs766325; rs6695765			NS		
*ROCK1*	18q11.1	rs35996865	Italy, Iran	189 triads	0.006	0.63 (0.45–0.88)	Palmieri et al. (2020)
*SUMO1*	2q33.1	rs3769817	Ireland	293 cases – 902 controls	0.038	1.45 (1.06–1.99)	Carter et al. (2010)
		rs12470401			NS		
*TBX22*	Xq21.1	rs6523677; rs6621541; rs7055763; rs58147590; rs6621542; rs41307258; rs6621543	Brazil, Europe, North America	126 cases – 295 controls	NS		Pauws et al. (2009)
*TCOF1*	5q32-q33.1	rs2255796	Taiwan, Singapore, United States	81 triads	0.033	2.08	Sull et al. (2008)
		rs2748222			0.096	1.77	
		rs1864957			0.077	1.91	
		rs15251			0.007	2.88	
		rs15251; rs28372960; rs2569062	Brazil	107 triads	NS		Machado et al. (2016)
*TGFA*	2p13.3	*Taq* I polymorphism*; GGAA4D07	Iowa	62 cases – 251 controls	NS		Lidral et al. (1998)
		rs2166975	Lithuania	18 triads	0.045		Morkuniene et al. (2007)
			Ireland	293 cases – 902 controls	0.041	1.42 (1.05–1.42)	Carter et al. (2010)
		rs6743202	Baltic regions	104 cases – 606 controls	0.0467	1.356 (1.004–1.831)	Nikopensius et al. (2010)
		rs1058213; rs2166975; rs930655; rs1523305; rs2902345; rs377122	Brazil	53 cases – 285 controls	NS		Letra et al. (2012)
		*Taq* I polymorphism*	Brazil	28 triads	NS		Souza et al. (2012)
		rs2166975	Ireland	296 triads – 62 dyads – 15 NSCPO	0.047		Fan et al. (2013)
		c.3851T>C; c.3822G>A	China	62 cases – 150 controls	NS		Xu et al. (2014)

*^#^Further information about the molecular function, biological process and relative pathway is given in Supplementary Table S1.*

*^†^NS, not significant.*

*^‡^OR for homozygous variant genotype.*

^§^Submucous cleft palate only.

*Deletion of 4 bp in intron V, creating a Taq I site.

### Cell–Cell Adhesion

The epithelial adhesion molecule cadherin (CDH1) participates actively in the epithelial–mesenchymal transition, the developmental step when the epithelial cells at the palatal midline disappear, allowing mesenchymal continuity and palatal fusion. Alterations in the *CDH1* gene contribute to the complex events that drive orofacial cleft, as demonstrated for the onset of non-syndromic cleft lip with or without cleft palate (NSCL/P) (Letra et al., 2009). Some studies have recently hypothesized that this gene may also represent a risk factor for NSCPO (Song and Zhang, 2011; Rafighdoost et al., 2013; Song et al., 2017).

Another gene involved in cell adhesion and motility that is crucial for craniofacial development is *CRISPLD2*. It contains an LCCL domain, common to other proteins involved in cellular migration (Chiquet et al., 2007). *CRISPLD2* product has also been shown to be important for normal migration and differentiation of neural crest cells during early palate formation (Swindell et al., 2015). A significant association between allelic variants at rs1546124 and rs4783099 in the *CRISPLD2* gene and the risk for NSCPO, has been reported in case–control studies (Shen et al., 2011; Gowans et al., 2016; Messetti et al., 2017).

One of the genes that participate in the signaling mechanism required for normal palate development is *JAG2*, which encodes a ligand for Notch family transmembrane receptors. *JAG2* and *NOTCH1* are spatio-temporally activated in the oral epithelia during palate development for the correct cell adhesion of the elevated palatal shelves, preventing their premature adhesion to other oral tissues (Casey et al., 2006). Nevertheless, only few studies investigated a possible involvement of *JAG2* in the onset of NSCPO, with controversial results. *JAG2* collaborates with *IRF6* on the same molecular pathway during oral epithelial differentiation. This coordination is essential for the control of palatal adhesion and fusion competence (Richardson et al., 2009). *IRF6* plays an important role in the formation and maintenance of the oral periderm, the spatiotemporal regulation of which is essential to ensure appropriate palatal adhesion (Richardson et al., 2009). Mutations in *IRF6* cause two common forms of syndromic cleft, known as Van der Woude Syndrome (VWS) and popliteal pterygium syndrome (Kondo et al., 2002). The crucial role of *IRF6* in the genesis of NSCL/P has been widely demonstrated in several studies. Zucchero et al. (2004), in a vast NSCL/P sample study from different countries, first demonstrated that genetic variants in *IRF6*, underlying VWS, might also be involved in the etiology of isolated clefts. However, although a strong association between genetic variants at *IRF6* and NSCL/P risk was successively confirmed by independent studies (Blanton et al., 2005; Ghassibe et al., 2005; Scapoli et al., 2005; Srichomthong et al., 2005), the existing results regarding NSCPO are contradictory (Table 1). This could be due to the low power of the studies because of small sample sizes, but also to the widely demonstrated different etiology of NSCPO with respect to NSCL/P.

A number of scientific evidences suggest that *FOXE1* is one of the most consistent genetic factors in the NSCPO etiology. Mutations of *FOXE1* cause Bamforth-Lazarus, a recessive syndrome characterized by cleft palate and congenital hypothyroidism (Castanet et al., 2002). Mice lacking *Foxe1* express a similar phenotype (De Felice et al., 1998). The FOXE1 is a transcription factor expressed in the shelf epithelium of the secondary palate during the fusion and regulates both *MSX1* and *TGFB3*, which are required for proper palate formation (Venza et al., 2011). Various studies reported a strong association between *FOXE1* and both NSCL/P or NSCPO (Moreno et al., 2009; Nikopensius et al., 2011; Lammer et al., 2016). No common missense mutations in *FOXE1* can explain the association signal evidenced by different authors, suggesting the hypothesis of a common variant in a regulatory region (Lidral et al., 2015). Sequence analysis identified a novel non-coding polymorphism c.-1204C>G in the promoter of *FOXE1* in 11 Italian NSCPOs (Venza et al., 2009), while Srichomthong et al. (2013) identified five non-synonymous mutations, none of which was present in the control population.

In craniofacial development, *TGFA* is expressed during the fusion of the palatine shelves at the level of the medial edge epithelium. Evidence of the involvement of *TGFA* in NSCPO etiology was found by several authors investigating different populations, but not by others, as detailed in Table 1.

### Cell Proliferation

*TCOF1* actively participates in the formation of neural crests. Alterations of this gene cause development anomalies that lead to craniofacial malformations (Dixon et al., 2006). Indeed, *TCOF1* is a causative gene for Treacher Collins syndrome, characterized by hypoplasia of the facial bones, cleft palate, and middle and external ear defects. Sull et al. (2008) identified the SNP rs15251 variant as a possible risk factor for NSCPO.

The homeobox gene *MSX1* actively participates in cell proliferation, differentiation, and apoptosis, all crucial processes for normal embryonic development. This gene plays a critical role in the epithelial–mesenchymal interaction during the formation of craniofacial bones (Satokata and Maas, 1994). Many studies on animal models have amply demonstrated *MSX1* involvement in palatal, facial and dental development (Satokata and Maas, 1994; Jumlongras et al., 2001). Genetic studies conducted on humans have ascertained a specific role for *MSX1* in the orofacial cleft (Jezewski et al., 2003; Modesto et al., 2006; Tasanarong et al., 2019). However, the association studies specifically dedicated to NSCPO cohorts obtained conflicting evidence, possibly due to the small sample sizes and the different ethnic groups considered (Table 1). A recent meta-analysis performed by Gu et al. (2018) of investigations published till 2017, indicated the SNP rs12532, able to affect mRNA expression and stability of *MSX1*, as a possible risk factor for NSCPO but not for NSCL/P.

*TBX22* encodes a transcription factor important for the mesenchymal proliferation and elevation of palatal shelves before their fusion. Its normal expression is related to a correct development of the palate, while *TBX22* mutations cause the hereditary X-linked disorder, cleft palate with ankyloglossia (CPX, OMIM 303400) (Marcano et al., 2004; Stanier and Moore, 2004). Marcano et al. (2004) screened for *TBX22* mutations in 238 NSCPOs from the Philippines, North America, and Brazil, and found 15 different variants: five mutations affecting the coding region, as well as several putative splice site mutations. Suphapeetiporn et al. (2007) by sequencing the *TBX22* gene in 53 NSCPO cases in the Thai population, found three missense mutations and a deletion in four affected individuals. Fu et al. (2015) reported a functional -73G>A mutation in the promoter of *TBX22*, in all five affected males of a six-generation Chinese family of NSCPO (Fu et al., 2015). Both *TBX22* and *MSX1* are post-translationally modified by SUMO1, a regulating factor that participates in the modulation of many other genes, with evidence of a role in human oral clefts (Dobreva et al., 2003; Ghioni et al., 2005; Lee et al., 2006; Andreou et al., 2007).

Shi et al. (2009) identified a *de novo* deletion on chromosome 2 in a Danish child affected by NSCPO. The estimated size of the deletion is 124.2 kb and contains *SUMO1* (Shi et al., 2009). In a large Irish study population composed by 383 NSCPO patients, the SNP rs3769817 located in *SUMO1* intron 2 was associated with an increased risk for cleft palate (Carter et al., 2010).

The PAX transcription factors are critical for neural crest induction (Monsoro-Burq, 2015). In particular, Pax7 regulates the cell cycle of embryonic stem cells and mouse embryonic fibroblasts (Czerwinska et al., 2016). The intronic rs742071 *PAX7* polymorphism, previously proposed as a risk factor for NSCL/P (Ludwig et al., 2012), was also found significantly associated with NSCPO in a family-based association study carried out with 266 European triads (Bohmer et al., 2013). The association with NSCPO was then confirmed in the Chinese and sub-Saharan African populations (Duan et al., 2017; Butali et al., 2019).

Another transcriptional factor widely involved in the processes of closure of the neural tube and craniofacial development is encoded by *GRHL3*. In families that do not show mutations at the *IRF6* locus, *GRHL3* seems to be the second candidate gene in VWS (Peyrard-Janvid et al., 2014). Functional analyses demonstrate that *GRHL3* is a target of IRF6 in the processes of periderm differentiation (de la Garza et al., 2013). In a genome-wide association study, Leslie et al. (2016) identified an association between the rs41268753 SNP and NSCPO in two independent populations. The importance of *GRHL3* in NSCPO etiology was independently confirmed by Mangold et al. (2016), who collected evidences supporting the non-synonymous polymorphism rs41268753 as a susceptibility factor for NSCPO and described rare mutations of this gene in patients. *COL2A1* is a cartilage-specific marker involved in cranial neural crest differentiation by complex mechanisms that involve epithelial–mesenchymal transition, migration, and differentiation (Ghassibe-Sabbagh et al., 2011). Mutations in genes coding for cartilage collagens II cause syndromes that are often associated with cleft palate or micrognathia, such as Pierre-Robin sequence and Stickler syndrome (Kannu et al., 2012). Evidence of an association between *COL2A1* alleles and NSCPO risk was found (Nikopensius et al., 2010), though not confirmed by others (Machado et al., 2016).

### Cell Migration

The two Rho kinase isoforms (ROCK1 and ROCK2) play essential roles in fundamental cellular processes such as contraction, adhesion, migration, apoptosis, and proliferation. ROCK regulates stress fiber and focal adhesion assembly (Phillips et al., 2012) and modulates cytoskeleton organization by phosphorylating different substrates, mainly myosin light chain and myosin phosphatase, involved in the contractility pathway that leads to normal palate development. A recent family-based association study carried out in two cohorts from Italy and Iran, showed a significant level of association between *ROCK1* rs35996865-G and NSCPO (Palmieri et al., 2020). Currently, there are no reports demonstrating the involvement of *ROCK2* in the onset of orofacial cleft, however, some studies show that the loss of both chromosome 18q and 2p (where *ROCK1* and *ROCK2* reside, respectively) determines a series of anomalies including defects of the palate and micrognathia (Suzuki et al., 2016).

FLNB belongs to a family of actin-binding proteins. Filamins are also able to interact with receptors and intracellular proteins involved in cytoskeleton-dependent cell proliferation, differentiation, and migration (Hu et al., 2014). Mutations in this gene cause malformations that include cleft palate as a feature, as reported by Jugessur et al. (2010) who suggest a maternal effect for variants mapping in this gene, in their NSCPO cohort (Jugessur et al., 2010).

### Genes Involved in Folate and Homocysteine Metabolism

Vitamin B9 or folate is not synthesized by our organism but is present, for example, in legumes, leafy green vegetables, some fruits, and eggs. Folic acid is the most stable form of folate, synthetically produced and usually used in vitamin supplements and fortified foods (Bailey et al., 2015). Adequate folate intake is essential for cell division and homeostasis. Indeed, the folate pathway plays a crucial role in several strategic biochemical processes such as DNA biosynthesis, methionine regeneration, amino acid metabolism, mitochondrial protein translation, and DNA methylation. Folate deficiency and/or aberrant folate metabolism during embryogenesis were therefore assumed to cause congenital malformations because of the alteration of the precise interplay of cell proliferation and death, migration, and differentiation needed in this delicate moment of development. However, as above documented, there is no strong association between folate supplementation during the periconceptional period and a decreased risk of having an NSCPO baby (Johnson and Little, 2008). Nevertheless, the folate pathway, with its enzymes and substrates, was suspected to have a role in the NSCPO etiology and several studies investigated associations between polymorphisms in genes related to one-carbon metabolism and cleft palate risk. The most investigated gene is *MTHFR*, with its C677T polymorphism. However, in a meta-analysis based on five studies, with a total of 576 CP cases and 2587 controls, no statistical significances were observed for the risk of NSCPO for heterozygotes, neither for homozygotes (Luo et al., 2012).

Table 2 reports information on folate and homocysteine metabolism gene polymorphisms investigated for their potential association with cleft palate, published until June 2020 in PubMed. Only single studies with data from a cohort or a subset of non-syndromic cleft palate cases have been considered in the table.

**TABLE 2:** Association studies between polymorphisms of one-carbon metabolism genes and NSCPO.

**Gene^#^**	**Locus**	**SNP information**	**Country**	**Sample size**^†^	***P*-value**^‡^	**OR (95% CI)**	**References**
*AHCY*	20q11.22	rs819142; rs819133	Norway	93 triads	NS		Boyles et al. (2009)
*BHMT*	5q14.1	rs3733890 – G742A – R239Q	Norway	191 triads	NS		Boyles et al. (2008)
			Norway	93 triads	0.022^§^		Boyles et al. (2009)
		rs567754; rs585800	Norway	93 triads	NS		Boyles et al. (2009)
*BHMT2*	5q14.1	rs626105	Caucasians, Hispanics, others	236 cases – 201 controls		0.9 (0.6–1.3)	Zhu et al. (2005)
		rs542721; rs10944	Norway	93 triads	NS		Boyles et al. (2009)
*CBS*	21q22.3	844ins68	Norway	191 triads	NS		Boyles et al. (2008)
			Norway	93 triads	NS		Boyles et al. (2009)
		rs234706 – C699T	Norway	191 triads	NS		Boyles et al. (2008)
			Norway	93 triads	NS		Boyles et al. (2009)
		rs234705; rs234709; rs4920037	Norway	93 triads	NS		Boyles et al. (2009)
		rs2124459	France	125 cases – 145 controls	0.02	0.61 (0.40–0.93)	Goffinet et al. (2016)
			Belgium	79 cases – 225 controls	0.02	0.64 (0.44–0.93)	Goffinet et al. (2016)
		rs4920037	Italy	129 triads	NS	1.18 (0.74–1.88)	Carinci et al. (2019)
			Iran – Tibet – Bangladesh	65 triads	NS	1.00 (0.48–2.10)	Carinci et al. (2019)
*CTH*	1p31.1	rs681475; rs1145920; rs515064; rs1021737	Norway	93 triads	NS		Boyles et al. (2009)
		rs663649	Norway	93 triads	0.047^§^		Boyles et al. (2009)
*DHFR*	5q14.1	rs2618372; rs380691	Norway	93 triads	NS		Boyles et al. (2009)
*DMGDH*	5q14.1	rs250513; rs479405; rs642013; rs2034899; rs1805074; rs248386; rs185077; rs532964	Norway	93 triads	NS		Boyles et al. (2009)
*FOLH1*	11p11.12	rs6485963; rs11040270; rs7113251; rs202720; rs10839236; rs202676	Norway	93 triads	NS		Boyles et al. (2009)
*FOLR1*	11q13.4	rs3016432; rs11235468	Norway	93 triads	NS		Boyles et al. (2009)
*FOLR2*	11q13.4	rs514933	Norway	93 triads	NS		Boyles et al. (2009)
		rs2298444			0.003		
*FOLR3*	11q13.4	rs533207; rs555306; rs575341	Norway	93 triads	NS		Boyles et al. (2009)
*FTCD*	21q22.3	rs1980983; rs9978174; rs4819208; rs2277820	Norway	93 triads	NS		Boyles et al. (2009)
*FTHFD*	3q21.3	rs1127717; rs3772430; rs2290053; rs2365004; rs2886059; rs1823213	Norway	93 triads	NS		Boyles et al. (2009)
*GNMT*	6p21.1	rs2296805; rs2274514	Norway	93 triads	NS		Boyles et al. (2009)
*MAT1A*	10q22.3	rs1556894; rs2993763; rs9285726	Norway	93 triads	NS		Boyles et al. (2009)
*MAT2A*	2p11.2	rs6739015; rs2028900; rs2028898; rs7568458	Norway	93 triads	NS		Boyles et al. (2009)
*MAT2B*	5q34	rs10515861; rs4869087; rs4869089; rs729352	Norway	93 triads	NS		Boyles et al. (2009)
*MTHFD1*	14q23.3	rs2236225 – G1958A – R653Q	Norway	191 triads	NS		Boyles et al. (2008)
			Ireland	321 triads	NS	1.31 (0.94–1.82)	Mills et al. (2008)
			Ireland	321 mothers – 1599 controls	0.02^§^	1.41 (1.05–1.09)	Mills et al. (2008)
			Norway	93 triads	NS		Boyles et al. (2009)
			Europe	242 triads	NS	0.93 (0.55–1.57)*	Mossey et al. (2017)
		rs3783731; rs8003379; rs1950902; rs2236224; rs1256146	Norway	93 triads	NS		Boyles et al. (2009)
*MTHFD2*	2p13.1	rs1667627; rs702462	Norway	93 triads	NS		Boyles et al. (2009)
*MTHFR*	1p36.3	rs1801133 – C677T – A222V	California	117 cases – 383 control	NS	0.8 (0.5–1.2)	Shaw et al. (1999)
			Norway	63 triads		2.4 (1.2–4.6)	Jugessur et al. (2003b)
			France	56 triads	NS	1.31 (0.6–3.0)	Chevrier et al. (2007)
			Norway	191 triads	NS		Boyles et al. (2008)
			UK	47 triads + 19 dyads^¥^		0.9 (0.44–1.77)	Little et al. (2008)
			Ireland	321 triads	NS	0.96 (0.64–1.43)	Mills et al. (2008)
			Ireland	321 mothers – 1599 controls	0.03^§^	1.5 (1.05–2.16)	Mills et al. (2008)
			Norway	93 triads	NS		Boyles et al. (2009)
			Africa	163 cases – 1078 controls	NS		Gowans et al. (2016)
			Europe	292 triads	NS	0.53 (0.31–0.92)*	Mossey et al. (2017)
			Italy	129 triads	NS	0.98 (0.68–1.42)	Carinci et al. (2019)
			Iran – Tibet – Bangladesh	65 triads	NS	1.17 (0.54–2.52)	Carinci et al. (2019)
		rs1801131 – A1298C – A429E	Norway	191 triads	NS		Boyles et al. (2008)
			Ireland	321 triads	NS	0.83 (0.49–1.40)	Mills et al. (2008)
			Norway	93 triads	NS		Boyles et al. (2009)
			Africa	163 cases – 1078 controls	NS		Gowans et al. (2016)
		rs4845877; rs1476413; rs3737964; rs12404124	Norway	93 triads	NS		Boyles et al. (2009)
*MTHFS*	15q25.1	rs685487; rs6495452; rs2562744	Norway	93 triads	NS		Boyles et al. (2009)
*MTR*	1q43	rs1805087 – A2756G	Norway	191 triads	NS		Boyles et al. (2008)
			Norway	93 triads	NS		Boyles et al. (2009)
		rs10925235; rs16834521	Norway	93 triads	NS		Boyles et al. (2009)
*MTRR*	5p15.31	rs1801394 – A66G	Norway	191 triads	NS		Boyles et al. (2008)
			Norway	93 triads	NS		Boyles et al. (2009)
		rs1532268	Norway	93 triads	0.021^§^		Boyles et al. (2009)
		rs3776455			0.038^§^		
		rs162031; rs162036; rs10380			NS		
*NOS3*	7q36.1	rs1800779 – A(-922G)	California	99 cases – 588 controls		0.9 (0.6–1.4)	Shaw et al. (2005)
		rs1799983 – G894T – E298D				1.1 (0.7–1.7)	
*PON1*	7q21.3	rs662 – A575G	Norway	191 triads	NS		Boyles et al. (2008)
			Norway	93 triads	NS		Boyles et al. (2009)
		rs854547; rs8491; rs854549; rs2237582; rs3917498; rs2074351; rs854565; rs2299261; rs705382	Norway	93 triads	NS		Boyles et al. (2009)
*SHMT1*	17p11.2	rs2168781	Norway	93 triads	0.015		Boyles et al. (2009)
		rs7207306			NS		
*SHMT2*	12q13.3	rs7311958	Norway	93 triads	NS		Boyles et al. (2009)
*SLC19A1/RFC1*	21q22.3	rs1051266 – A80G	California	123 cases – 364 controls		0.8 (0.5–1.3)	Shaw et al. (2003)
			Norway	191 triads	NS		Boyles et al. (2008)
			Norway	93 triads	NS		Boyles et al. (2009)
*SLC46A1*	17q11.2	rs9894260	Utah	109 triads	NS	0.75 (0.32–1.78)	VanderMeer et al. (2016)
		rs739439			NS	1.40 (0.62–3.15)	
		rs2239907			NS	0.73 (0.42–1.27)	
*TCN2*	22q12.2	rs1801198 – C776G – P259R	Norway	191 triads	NS		Boyles et al. (2008)
			Ireland	321 triads	NS	1.05 (0.73–1.52)	Mills et al. (2008)
			Norway	93 triads	NS		Boyles et al. (2009)
			Italy	129 triads	NS	0.98 (0.69–1.40)	Carinci et al. (2019)
			Iran – Tibet – Bangladesh	65 triads	NS	1.10 (0.60–2.02)	Carinci et al. (2019)
		rs9606756 – A67G	Norway	191 triads	NS		Boyles et al. (2008)
			Norway	93 triads	NS		Boyles et al. (2009)
*TYMS*	18p11.32	rs502396; rs2244500; rs10502290; rs10502289	Norway	93 triads	NS		Boyles et al. (2009)
		rs16430 – 1494del6	California	123 cases – 581 controls		1.3 (0.8–2.0)	Shaw et al. (2013)
		rs45445694 – 28-bp VNTR	California	123 cases – 581 controls	Significant	1.8 (1.1–3.1)*	Shaw et al. (2013)

*^#^Further information about the molecular function, biological process and relative pathway is given in Supplementary Table S2.*

*^†^Studies with a cohort bigger than 50 cases are reported.*

*^‡^NS, not significant.*

^§^When the maternal association is considered.

*OR for homozygous variant genotype.

*^¥^In the sample study are included 11 cases of Pierre Robin sequence.*

### Genes Responsible for Syndromic Forms as Candidate for NSCPO

As mentioned above, referring to Burg et al.’s (2016) work, the responsible genes have been identified in a number of syndromes that include cleft palate among their features. Based on the assumption that genes implicated in syndromic forms of cleft could also have a role in non-syndromic phenotypes, the variant association of such genes has been questioned in case control and family based studies on different NSCPO worldwide cohorts.

The attempts of researchers to confirm this hypothesis are summarized in Table 3, where genes already mentioned in a previous section (*FLNB*) or in Table 1 (*TBX22*, *FOXE1 COL2A1*, *IRF6*, *TCOF1*) are omitted.

**TABLE 3:** Genes responsible for syndromic cleft palate, studied in NSCPO cohorts.

**Gene^#^**	**Locus**	**Syndrome with cleft palate**	**SNP information**	**Country**	**Sample size**	***P* value**^†^	**OR (95% CI)**	**References**
*BCOR*	Xp11.4	Oculofaciocardiodental	Haplotype rs4076107-rs6520620-rs5963158-rs4308866	Asia	253 triads	<10^–4‡^		Skare et al. (2017)
			rs6609051; rs12687359	Norway – Denmark	114 + 69 triads	NS		Jugessur et al. (2012)
*COL11A1*	1p21.1	Pierre Robin Sequence and Stickler	42 genotyped SNPs	Baltic regions	104 cases – 182 controls	NS		Nikopensius et al. (2010)
*COL11A2*	6p21.32	Pierre Robin Sequence and Stickler	rs213209	Baltic regions	104 cases – 182 controls	0.0138	0.63 (0.435–0.912)	Nikopensius et al. (2010)
			rs9277928			0.0451	0.637 (0.408–0.993)	
*EFNB1*	Xq13.1	Craniofrontonasal	rs877818	Norway – Denmark	114 + 69 triads	NS		Jugessur et al. (2012)
*FGFR1*	8p11.23	Kallman type 2	rs2978083	Baltic regions	104 cases – 606 controls	0.014	0.3 (0.109–0.829)	Nikopensius et al. (2010)
			rs7829058			0.0049	1.789 (1.189–2.720)	
				Brazil	41 triads	NS		Machado et al. (2016)
*FGFR2*	10q26.13	Apert and Crouzon	rs1047100	Ireland	293 cases – 902 controls	NS	0.85 (0.64–1.14)	Carter et al. (2010)
*FLNA*	Xq28	Otopalatodigital spectrum disorders	rs766419; rs2070822; rs2070816	Norway – Denmark	114 + 69 triads	NS		Jugessur et al. (2012)
*OFD1/CXORF5*	Xp22.2	Oral-facial-digital syndrome 1	rs2285635; rs2283707	Norway – Denmark	114 + 69 triads	NS		Jugessur et al. (2012)
*PHF8*	Xp11.22	Siderius type	rs6521788; rs12115965; rs7876951; rs5960612	Norway – Denmark	114 + 69 triads	NS		Jugessur et al. (2012)
*PQBP1*	Xp11.23	Renpenning	rs4824733; rs2016813; rs741932	Norway – Denmark	114 + 69 triads	NS		Jugessur et al. (2012)
*SMS*	Xp22.11	Snyder-Robinson	rs2040357; rs5951678	Norway – Denmark	114 + 69 triads	NS		Jugessur et al. (2012)
*SOX9*	17q24.3	Pierre Robin sequence and Campomelic dysplasia	rs12941170	China	46 triads	0.03	0.56 (0.3–0.96)	Jia et al. (2017)
			rs2229989			0.06	0.57 (0.3–1.03)	
*TGFB2*	1q41	Loeys-Dietz	pPC-27 probe	Philippines	48 cases – 214 controls	NS		Lidral et al. (1997)

*^#^Further information about the molecular function, biological process and relative pathway is given in Supplementary Table S3.*

*^†^NS, not significant.*

*^‡^Association among 100 males.*

### Genome-Wide Analysis

The rapid improvement, in recent years, of technological tools for genotyping and sequencing has allowed researchers to approach whole genome studies. The aim is to identify as many susceptibility genes as possible, while the challenge is to collect and analyze dataset large enough to deliver the required statistical power. The genome-wide association studies (GWAS) approach is an indirect mapping technique, able to identify associated polymorphisms, while exome/genome sequencing (WES/WGS) studies are more focused on rare genetic mutations. Table 4 reports genes and polymorphisms associated with NSCPO detected by GWAS. Besides *IRF6*, an already known genetic risk factor for orofacial clefts, the list includes several new candidate genes. Among these *GRHL3*, whose involvement in NSCPO etiology was confirmed in an independent European cohort (Leslie et al., 2016) and by Mangold et al. (2016) in a subsequent investigation in which they evidenced common and rare causative mutations.

**TABLE 4:** Genome wide association studies in NSCPO cohorts.

**SNP information**	**Locus**	**Gene^#^**	**Country**	**Sample size**	***P*-value**^†^	**OR (95% CI)**	**References**
			49% Europe – 47% Asia – 3% Africa	550 triads	NS		Beaty et al. (2011), Shi et al. (2012), and Ludwig et al. (2017)
rs41268753	1p36.11	*GRHL3*	Europe	38 cases – 93 – triads – 835 controls	4.08 × 10^–9^	8.3 (1.17–59.15)	Leslie et al. (2016)
rs117496742	11q22.1	*YAP1*	Europe	38 cases – 93 – triads – 835 controls	3.13 × 10^–8^	11.2 (1.04–121.8)	Leslie et al. (2016)
rs12175475	6p26	*PARK2*	Multiethnic	78 cases – 165 triads – 1700 controls	8.66 × 10^–9^	6.6 (1.12–39.58)	Leslie et al. (2016)
rs80004662	2p12	*CTNNA2*	Sub-Saharan Africa	205 cases – 2159 controls	7.41 × 10^–9^	7.5 (3.45–16.28)	Butali et al. (2019)
rs730570	14q32.2	*DLK1*	China	2071 cases – 10145 controls	6.59 × 10^–10^	1.28	Huang et al. (2019)
rs4646211	13q32.3	*DOCK9*	China	2071 cases – 10145 controls	4.78 × 10^–12^	1.28	Huang et al. (2019)
rs8061677	16q24.2	*FOXC2-FOXL1*	China	2071 cases – 10145 controls	9.11 × 10^–11^	1.29	Huang et al. (2019)
rs72741048	1q32.2	*IRF6*	China	2071 cases – 10145 controls	3.07 × 10^–15^	1.314	Huang et al. (2019)
rs1009136	19p13.11	*MAU2*	China	2071 cases – 10145 controls	2.66 × 10^–9^	1.25	Huang et al. (2019)
rs730643	14q13.3	*PAX9*	China	2071 cases – 10145 controls	2.92 × 10^–16^	0.74	Huang et al. (2019)
rs6791526	3p22.1	*POMGNT2*	China	2071 cases – 10145 controls	1.62 × 10^–10^	1.46	Huang et al. (2019)
rs3468	4p16.3	*WHSC1*	China	2071 cases – 10145 controls	5.4 × 10^–11^	1.256	Huang et al. (2019)

*^#^*Further information about the molecular function, biological process and relative pathway is given in Supplementary Table S4*. *^†^NS, not significant SNP detected.**

Whole exome sequencing (WES) searching for NSCPO mutations was performed in affected individuals from multiplex families with NSCPO (Hoebel et al., 2017). A probable deleterious mutation in *ARHGAP29*, a gene expressed in developing palate, was found in four individuals of a single family affected by cleft of the soft palate (Liu et al., 2017). The WES of 16 individuals from 8 multiplex families allowed Hoebel et al. (2017) to select 26 candidate genes that were sequenced in additional 132 NSCPO cases. The investigation failed to identify genes with recurrent deleterious mutations but produced data that may be useful for subsequent investigations.

The potential role of epigenetic regulation in NSCPO has been recently explored by two groups (Sharp et al., 2017; Xu et al., 2019). In these investigations, epigenome-wide association studies (EWAS) were conducted to test whether DNA methylation of blood, or other relevant tissues, was associated with orofacial clefting. These preliminary investigations suggest differences in methylation profiles of patients and unaffected controls, as well as between different types of clefts. However, future result replications may be difficult because methylation status may consistently vary as a function of kind of investigated tissue, and of patient age at the moment of sampling.

### Gene × Gene Interaction

Gene–gene epistatic interaction (GxG) analysis has been successfully adopted for other diseases in order to narrow a critical region (Cox et al., 1999) or to identify additional loci (Pankratz et al., 2003). A small number of researchers tested for GxG in cohorts that do not include syndromic forms of CPO nor patients with CL. In detail, Jugessur et al. (2003a) found a significant 6.3-fold increased risk of NSCPO in patients carrying the *TGFA Taq* I A2A2 genotype in combination with one or two copies of the T-allele at *MTHFR* C677T polymorphism. This same gene–gene interaction was investigated by Mossey et al. (2017), evidencing an opposite effect when considering the combination *Taq* I A2 and one or two copies of *MTHFR* variant for the child genotype, with a 6 and 12.5-fold reduced risk, respectively. Lastly, Duan et al. (2017) evidenced an epistatic interaction between rs4844913 (43 kb 3′ of *DIEXF*) and rs11119388 (*SYT14*) and between rs6072081 (53 kb 3′ of *MAFB*) and rs6102085 (33 kb 3′ of *MAFB*).

### Gene × Environment Interactions

Orofacial clefts, including NSCPO, are considered as typical complex diseases caused by several genetic and environmental factors. Although many candidate gene studies and GWAS have been performed to reveal genetic factors of NSCPO, the genetic variants identified so far can explain only a small fraction of heritability of this common malformation. This has opened the hypothesis that the effect of genetic factors on a disorder can be modulated by environmental factors and *vice versa*. Several different models of gene–environment interactions (GxE) were postulated; in some cases it could be easier to detect the effect of genetic factors if the effect of environmental factors is also considered (Ottman, 1996).

The first attempts to investigate GxE interaction in OFC were reviewed by Murray (2002). Most of these studies included NSCL/P cases only, or NSCL/P cases together with a small fraction of NSCPO cases. Some possible GxE interactions were suggested but not confirmed (Murray, 2002).

An interaction between the rs7205289 polymorphism and passive smoking in NSCPO was observed in the Chinese population (Li et al., 2011). The rs7205289 lies in the microRNA-140 gene and can reduce its expression (Li et al., 2011). Interestingly, tobacco smoke had the same effect in cultured mouse palatal mesenchymal cells (Li et al., 2011). This data is particularly relevant because dysregulation of miRNA-140 was found to cause palatal malformations in zebrafish by targeting *Pdgfra* (Eberhart et al., 2008; Li et al., 2011). The *Pdgfra* has a critical role in neural crest development, mesenchymal cell migration, and palatogenesis also in mice (McCarthy et al., 2016). Finally, mutations in *PDGFRA* were found in NSCPO patients (Rattanasopha et al., 2012).

In a GxE study on OFC cases, Shi et al. (2007) investigated the interaction between maternal smoking and 16 candidate genes involved in detoxification. An interaction was found with *GSTT1* among OFC, with a higher effect in the NSCPO subgroup. In particular, the combination of fetal *GSTT1*-null genotype and maternal smoking provided the highest risk of cleft. This finding was important, because it confirmed a previous study involving an OFC sample from The Netherlands (van Rooij et al., 2001).

A GWAS conducted with 550 patient-parents triads supported the importance of a GxE interaction investigation. Indeed, while no individual gene reached the statistical threshold of significance in the test of association with NSCPO, several genes demonstrated an association when their interaction was considered. In particular, polymorphisms of *MLLT3* and *SMC2* interacted with maternal alcohol consumption; *TBK1* and *ZNF236* with maternal smoking; and *BAALC* with multivitamin supplementation (Beaty et al., 2011). Data were further examined by stratifying the sample by European and Asian ancestry (Wu et al., 2014). Several polymorphisms of a chromosome 4 region that includes *SLC2A9* and *WDR1* genes were associated with NSCPO in Asians when the interaction with environmental tobacco smoke exposure was tested.

The same dataset was investigated with an alternative model that includes the parent of origin (PoO) effect, i.e., the risk in the child varies on whether the allele is inherited from the mother or the father. The hypothesis of interaction between PoO and maternal exposure (PoOxE) provided the evidence for new genes involved in NSCPO etiology. Indeed, several polymorphisms in *ICE2* and *NAALADL2* showed PoOxSmoke interaction in European families (Haaland et al., 2017).

## Discussion

Defects of the closure of the secondary palate only are, among orofacial clefts, the rarest, the least studied, and those with the least obvious nature. CPO can occur as a feature of Mendelian single gene disorders or of several chromosomal syndromes. On the other hand, the isolated form of CPO is considered to have a multifactorial etiology, and in the last decades researchers have attempted to elucidate all the teratogenic and genetic factors causing this common malformation. The identification of causal factors and understanding of the molecular mechanisms of cleft formation would be an invaluable aid for clinicians in optimizing prevention approaches, providing genetic counseling, and planning personalized surgical and medical treatments.

Several evidences have highlighted the fact that NSCPO and NSCL/P are different malformations with different, or slightly overlapping, causes. Scientific research has been mainly focused on NSCL/P, while NSCPO study has been marginal, maybe owing to the lower incidence that makes harder to collect cohorts of comparable size. In recent years, this gap has been reducing, but new insights on CPO are accumulating more slowly than expected, especially with regards GWAS. A possible explanation is a limited contribution of common genetic variants in NSCPO etiology, which lower the success rate of association analyses. Genetic investigations have provided convincing evidences for a limited number of heritable factors that do not account for the high level of hereditability of NSCPO. These include *FOXE1*, *GRHL3*, and *PAX7*, while a number of investigations provided support for transcription and regulatory factors necessary for the normal development of neural crests. If rare variants play a major role in CPO etiology, future large-scale sequencing efforts may represent a promising approach to detect genetic factors. Emerging trends of research regard more complex models, including gene–gene interaction, gene–environment interaction, and epigenetic analyses, which could be more powerful to explain missing heritability, but all of these require larger sample sizes. However, such approaches need a deeper investigation, in order to earlier predict NSCPO risk and to plan healthcare strategies aimed at improving environmental conditions and reducing exposure to potential epigenetic factors.

Considering the long, but not exhaustive, list of potential susceptibility factors for NSCPO, a wide range prevention could appear unrealistic, also because palate closure occurs during a gestation period in which the mother is often unaware she is pregnant. It is in any case crucial to identify exposure risks acting around the conception time (1 month before, 3 months after) that may impact embryonic development. A consistent bulk of data supports an epidemiological role for both active and passive tobacco smoking, while contrasting evidences have been collected for moderate alcohol consumption. On the contrary, folate fortification and multivitamin supplementation seem to decrease the risk of NSCPO.

## Author Contributions

MM conceived the idea and design of the manuscript, acquired most of the references and data, and contributed to the drafting, revision, and final approval of the manuscript. AP and FC contributed to the manuscript design and critical revisions and final approval of the manuscript. LS contributed to the design, drafting, acquisition of statistical data, revision, and final approval of the manuscript. All authors contributed to the article and approved the submitted version.

## Conflict of Interest

The authors declare that the research was conducted in the absence of any commercial or financial relationships that could be construed as a potential conflict of interest.

## Footnotes

1 https://www.ncbi.nlm.nih.gov/omim
